# Correspondence

**Published:** 1879-02

**Authors:** 


ATALNTA

### Medical and Surgical Journal.

VOL. XVI.] FEBRUARY—1879. [No. 11

### Original Communications.

*Editor Atlanta Medical and Surgical Journal:*

Please insert the inclosed letter from Dr. Stanford, of Columbus, Ga.

There is no question but that the Doctor gives the history of the operation in America. In my remarks before the Academy of Medicine, reported in the October number of the JOURNAL, I did not intend to convey the idea that Dr. Pancoast was the first surgeon to remove the parotid gland, but mentioned the fact to show that as late as 1849 prominent surgeons doubted the possibility of its complete extirpation. Very truly, etc.,

W. F. WESTMORELAND.

*Dr. Willis F. Westmoreland, Professor of Surgery Atlanta Medical College:*

MY DEAR FRIEND—In the October number of the MEDICAL AND SURGICAL JOURNAL, published in your city, discoursing upon the operation for the extirpation of the parotid gland, before the Academy of Medicine, you are reported as saying that when you were a student "Dr. Pancoast successfully executed it, and presented the specimen to Dr. Gibson, demonstrating its feasibility."

That justice may be fully awarded to the surgeon to whom it is due, I trust, my dear Doctor, that you will permit me to place upon the record the result of my knowledge upon this subject.

The operation for the extirpation of the parotid gland was first performed in this country by Dr. Warren, of

Boston, Mass., in 1798. After this it slumbers, and we hear nothing more of it until 1826, when it was again performed by Dr. George McClellan, of the Jefferson School of Philadelphia. Dr. McC. successively performed this operation in eleven cases. Ten of these cases, including the first, were completely successful, ending in the restoration to health.

Dr. Wm. Gibson, of the University, the rival school to the Jefferson, denied that Dr. McClellan had removed this gland, alleging that it was one of the many lymphatics situated on the gland.

Professional excitement was worked up to the highest pitch between the friends of the rival schools, and personal feeling was much embittered.

Prof. Granville Sharpe Pattison, occupying the chair of Anatomy in the Jefferson school, demonstrated the entire feasibility of the operation—declaring that Dr. McClellan had removed the gland in its entirety, thereby giving to him the credit of establishing it as an operation to be performed.

Directly or indirectly, growing out of the controversy, a hostile meeting took place between Prof. Pattison and Gen. Cadwallader, who espoused the cause of Prof. Gibson.

A resident student of medicine myself, during the years 1845, 1846, 1847 and most of 1848, at the University of the city of New York, Prof. Pattison, then occupying the chair of Anatomy in that school, it was my pleasure often to listen to his demonstration of the practicability of the operation; and it was his delight to give all credit to Dr. McClellan.

This operation, soon after this, was performed by different surgeons, and I presume often by Dr. Pancoast.

Dr. Gibson confessed his error long before his death.

Dr. McClellan's first case was in the person of a medical student named Graham, who, after recovering from the operation, became a practitioner of medicine in New York.

Very respectfully your ob't. serv't,

F. A. Stanford, M.D.,

*Columbus, Ga., Dec.* 4, 1878.

